# Possibility of Establishing an Early Diagnosis in Cerebro-Spinal Meningitis

**Published:** 1916-03

**Authors:** 


**Possibility of Establishing an Early Diagnosis in Cerebro-Spinal Meningitis.** Gaceta Medica de Costa Rica, Aug. 15, 1915. Dr. J. Aguilar Jordán urges that more attention should be devoted to this subject. He divides the morbid processes into three periods:—

1. Catarrhal;
2. Septicemic;
3. Meningeal.

In the first stage there are: coryza, redness of the palate, with a fine punctuate eruption of a brighter hue, and occasionally vesicles. The pharynx is also erythematous; the voice is changed; there is hoarseness and cough. Conjunctivitis and herpes labialis present themselves. Fever is present and the reflexes are exaggerated. In the second stage the diplococcus is found in the blood and the urine; very abundantly in the latter according to Thomas and Fleming. Also a few meningococci, usually dead, are found in the cerebro-spinal fluid and much albumen. In suspected cases, the examination of the naso-pharyngeal secretions, the blood and the urine should be instituted to rule out grippe. He recommends the vaccine of Lundie in the first and second periods, and states that notable results ensue from the very first injection. Disinfection of the naso-pharyngeal region and the urine, with the complete isolation of the patient should be carried out. He lays stress on the fact that the cerebro-spinal symptoms represent the third and last stage of the disease, while but a few years ago these were considered to be the primary manifestations of the affection. To him, the symptoms of the primary stage are as characteristic as those presented by patients in the sleeping stage of trypanosomiasis.

